# Bronchobiliary fistula caused after hepatectomy for hepatocellular carcinoma: a case report

**DOI:** 10.1186/s40792-016-0273-z

**Published:** 2016-12-05

**Authors:** Seikan Hai, Yuji Iimuro, Tadamichi Hirano, Kazuhiro Suzumura, Akito Yada, Jiro Fujimoto

**Affiliations:** Department of Surgery, Hyogo College of Medicine, 1-1 Mukogawa-cho, Nishinomiya, Hyogo 663-8501 Japan

**Keywords:** Bronchobiliary fistula, Bile leakage, Hepatectomy

## Abstract

**Background:**

A bronchobiliary fistula, an intercommunication between the biliary tract and bronchial trees, is an extremely rare complication after hepatectomy.

**Case presentation:**

A 70-year-old male underwent partial resection of the liver for recurrent hepatocellular carcinoma under a thoracoabdominal approach. The immediate postoperative clinical course was uneventful, but the patient was febrile and laboratory examinations revealed leukocytosis on the 15th postoperative day. An intraabdominal abscess was suspected based on the computed tomography findings, and percutaneous drainage was performed. Bile was drained, and fluoroscopy using a contrast medium from the drainage tube revealed a communication between the cavity and the common hepatic duct. Two weeks after drainage, bilioptysis was seen. Fistulography demonstrated the presence of the bronchus in the right lower lobe of the lung via the subphrenic space. Therefore, the patient was diagnosed to have a bronchobiliary fistula. Fistulography revealed closure of the communication with the bronchus about a month after drainage. However, the bile leakage and bilioptysis did not stop even after endoscopic nasogastric biliary drainage, and ethanol injection therapy were performed. Eventually, residual right bisectionectomy without resection of the fistulous tract and involved lung was performed to remedy the intractable bile leakage. The clinical course after the reoperation was good without bile leakage, bilioptysis, or pulmonary disorders, and the patient was discharged 40 days after reoperation.

**Conclusions:**

We experienced a rare case of bronchobiliary fistula that occurred after hepatectomy for hepatocellular carcinoma. Careful attention should be paid to prevent bile leakage during hepatectomy, since bile leakage has the potential to cause a bronchobiliary fistula.

## Background

A bronchobiliary fistula (BBF), an intercommunication between the biliary tract and bronchial trees, is a rare condition. Several causes of BBF, including amoebic diseases of the liver, trauma, biliary obstruction, and hepatobiliary surgery, have been reported, and appropriate treatment is required, because a BBF can be associated with high mortality and morbidity rates [1]. We herein present a case required reoperation for BBF with intractable bile leakage that occurred after partial hepatic resection for hepatocellular carcinoma.

## Case presentation

**Fig. 1:**
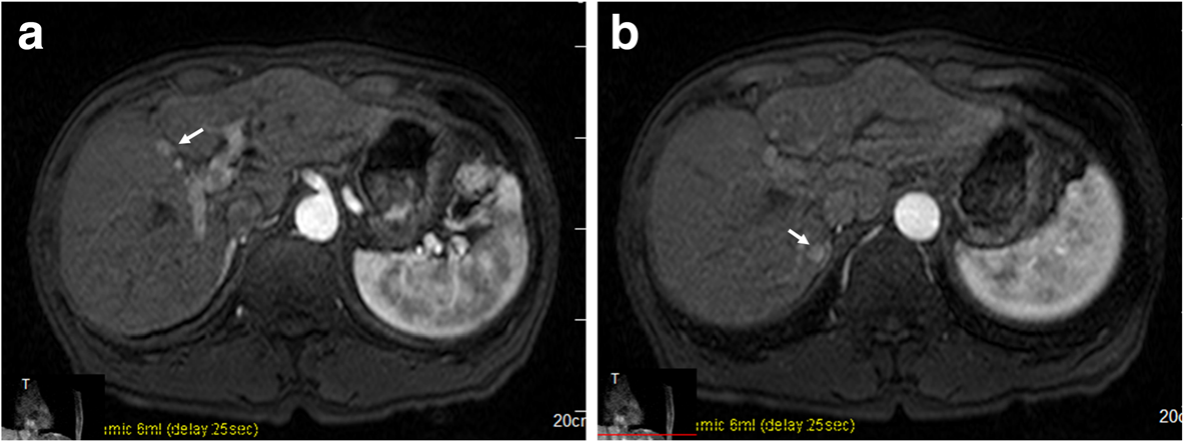
Magnetic resonance imaging shows a mass lesion enhanced during the early phase of contrast enhancement in segment 5 (**a**) and segment 7 (**b**)

**Fig. 2:**
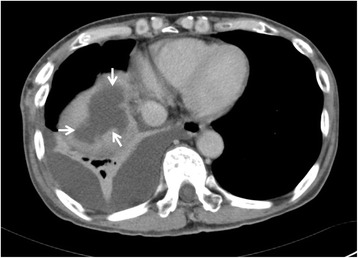
Computed tomography shows a low-density lesion (*arrows*) at the cut surface of the liver in the subphrenic space

**Fig. 3:**
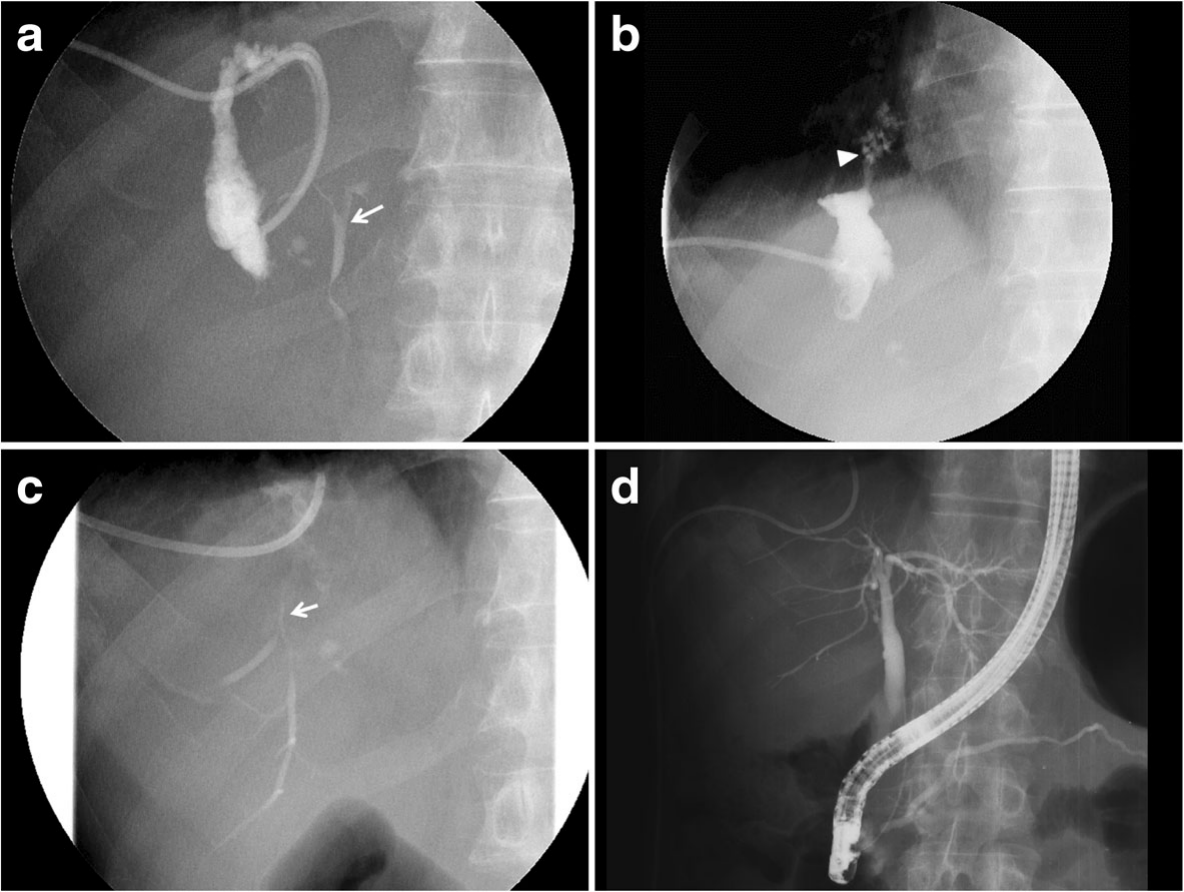
**a** A fistulogram shows an abscess cavity communicating with the common bile duct (*arrow*). **b** Two weeks after drainage, fistulography demonstrates the presence of the bronchus (*arrowhead*) in the right lower lobe of the lung via the subphrenic space. **c** The peripheral bile duct (*arrow*) in segment 5 of the liver is seen by fistulography 3 months after drainage. **d** The injured bile duct in segment 5 is not demonstrated by endoscopic retrograde cholangiography

A 70-year-old male had undergone partial resection of the liver for hepatocellular carcinoma (HCC) in segment 8 under a thoracoabdominal approach 9 years earlier. During a routine follow-up, magnetic resonance imaging showed two low-intensity masses in segments 5 and 7 of the liver on T1-weighted imaging, which were enhanced during the early phase of contrast enhancement (Fig. 1a, b). We made a diagnosis of recurrent HCC and performed partial resection under thoracoabdominal approach. The immediate postoperative clinical course was uneventful, and the drains were removed on postoperative day (POD) 8. On POD 15, the patient was febrile and laboratory examinations revealed leukocytosis (10,500/μl; normal range, 4000 to 9000/μl) and an increased serum concentration of C-reactive protein (CRP) (9.5 mg/dl; normal range, 0 to 0.3 mg/dl). Abdominal computed tomography (CT) demonstrated fluid collection in the space near the cut surface and retained pleural effusion (Fig. 2), and percutaneous drainage was intercostally performed with a 7.2-Fr pigtail catheter. At that time, bile was found in the drain fluid, and *Staphylococcus epidermidis* was detected from the discharge. The pleural effusion was drained 10 days after abscess drainage. The pleural effusion did not contain bile or bacteria. Fluoroscopy using a contrast medium from the drainage tube revealed a communication between the cavity and the common hepatic duct (Fig. 3a). Two weeks after the abscess drainage, expectoration of bile-stained sputum, so-called bilioptysis, was seen, and fistulography demonstrated the presence of the bronchus in the right lower lobe of the lung via the subphrenic space (Fig. 3b). Therefore, the patient was diagnosed to have a BBF. Continuous suction drainage provided relief from the bilioptysis, and fistulography revealed no communication with bronchus about a month after drainage.

However, bile continued to be discharged at a rate of 50 to 100 ml per day, and it became clear that this was caused by an injured bile duct in segment 5 of the liver by fistulography and drip infusion cholangiography-CT 3 months after the initiation of drainage. At that time, the hilar side of the bile duct could not be demonstrated by fistulography (Fig. 3c), and the periphery of the injured bile duct could also not be demonstrated by endoscopic retrograde cholangiography (ERC) (Fig. 3d). ERC revealed no stricture of the common hepatic duct or common bile duct. Based on these findings, the bile leakage in this patient was thought to have lapsed into the interrupted type due to continuous drainage.

**Fig. 4:**
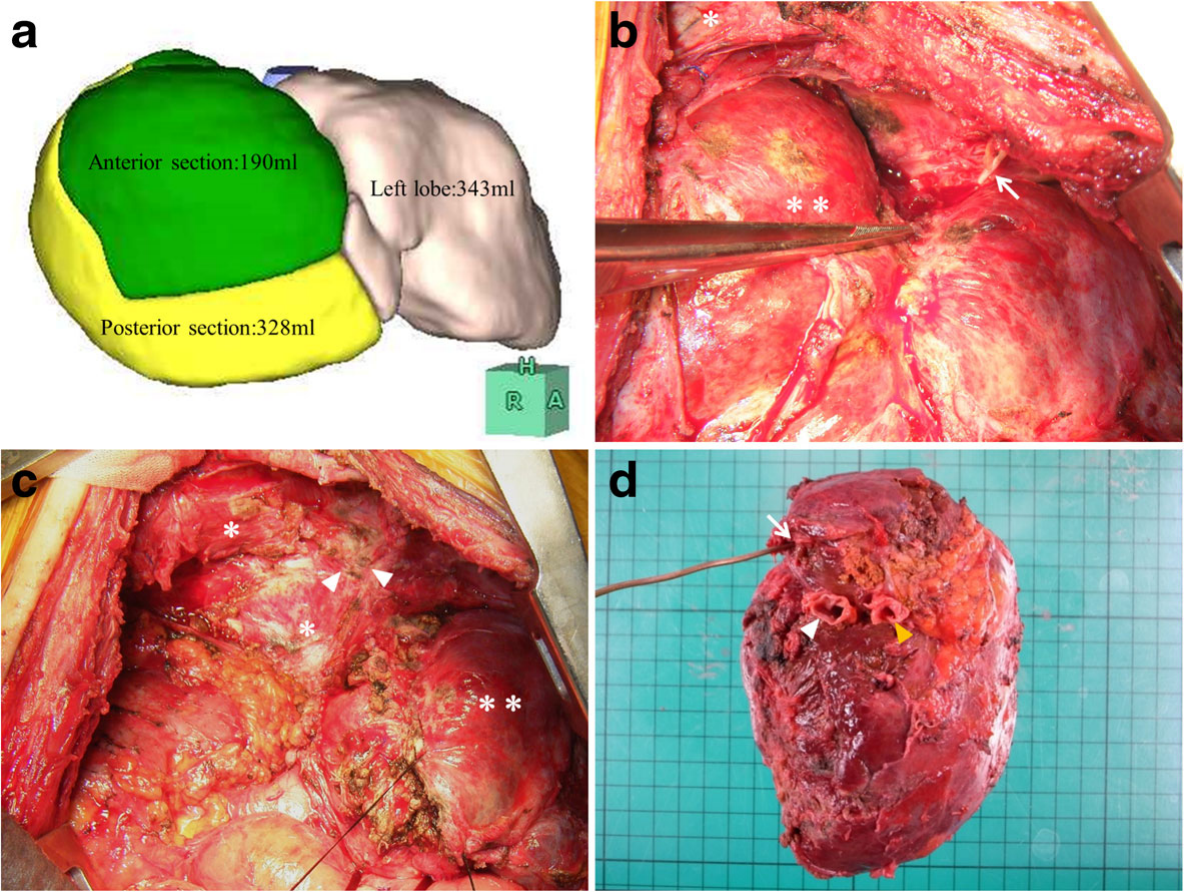
**a** Volumetric analysis. **b** At laparotomy, a drainage tube (*arrow*) is inserted into the abscess cavity, and the lower lung (*asterisk*) and the remnant right lobe of the liver (*asterisks*) found to be strongly adherent to the diaphragm. **c** A fibrous scar, which is thought to be the BBF (*arrowheads*), is seen on the sutured line of the diaphragm (*asterisk*); (*asterisks*: left lobe of the liver) **d** Resected specimen. Weight of resected liver was 499 g and stump of the right hepatic vein (*arrow*), the anterior branch of the portal vein (*white arrowhead*), and the posterior branch of the portal vein (*yellow arrowhead*) are seen

Predictably, endoscopic nasogastric biliary drainage (ENBD) could not stop the bile leakage. Thereafter, we performed ethanol injection (1 to 1.5 ml 95% ethanol) into the peripheral bile duct three times because a drainage tube could form an external fistula with the injured peripheral bile duct, but the amount of bile discharged from the drainage tube could not be decreased. In addition, bilioptysis was often seen when continuous suction drainage stopped, despite bronchus was not demonstrated by fistulography. Seven months have passed after drainage, intractable bile leakage and BBF could not heal only by percutaneous drainage, so we decided to perform reoperation after obtaining informed consent. Residual anterior sectionectomy, including the injured bile duct in segment 5, was initially considered because the operative procedure was thought to have a low risk of postoperative hepatic failure based on the ratio of the estimated hepatic resection volume (22.1%). However, it was considered that the anatomical resection of the residual anterior section would be difficult and that there would be a risk of causing new bile leakage due to the presence of dense adhesions and the anatomical modifications of the liver in the second previous hepatectomy. For these reasons, residual right bisectionectomy was eventually planned to treat the intractable bile leakage without fail based on preserved liver function tests (Child-Pugh A, indocyanine green retention at 15 min: 12%) although the ratio of estimated hepatic resection volume was 60.1% by hepatic volumetric analysis (Fig. 4a). The reoperation was again performed under a thoracoabdominal approach, but between different ribs. When the dense adhesions between the liver and diaphragm were broken up, bile containing a pus-like discharge flowed out, and the tip of the drainage tube was inserted into the cavity (Fig. 4b). The source of the bile leakage could not be identified. A fibrous scar, which was thought to form the BBF, was seen on the sutured line of the diaphragm during the last operation, and adhesions were present between the lower lung and the diaphragm. Residual right bisectionectomy without resection of the fistulous tract and involved lung was performed as planned (Fig. 4c, d). The clinical course after the reoperation was good, without hepatic failure, bile leakage, bilioptysis, or pulmonary disorders, excluding ascites that required the administration of diuretics, and the patient was discharged 40 days after the reoperation. As of 4 years after discharge, the patient is doing well without any signs of BBF recurrence.

### Discussion

A bronchobiliary fistula is a rare condition that was first reported in a patient with hydatid disease of the liver in 1850 [2]. The major causes of BBF vary by area, with echinococcal and amoebic diseases of the liver being the most common in developing countries, and trauma and biliary obstruction being the most common in Western countries [1]. Although rare, BBFs have been reported in English literatures to develop after treatments for HCC [3–13] (Table 1). In almost all of the cases, BBF occurred after transcatheter arterial chemoembolization and/or radiofrequency ablation for HCC; there were only two reported cases of a BBF occurring after hepatic resection. In the one previous report in which a BBF occurred after hepatic resection for HCC [5], the authors suggested that a biliary tract injury during hepatic resection caused biliary stenosis and bile leakage and that a subphrenic abscess caused by biliary stenosis eroded the diaphragm and directly perforated the bronchial system. In another report, it was speculated that a BBF was iatrogenic because the authors discussed that percutaneous transhepatic biliary drainage for strictures of common bile duct occurred after central lobectomy for HCC was the possible etiology of BBF [9]. The pathogenesis of a BBF is generally considered to include the following: the mechanical effects of increased intraluminal pressure on the biliary system due to obstruction of the bile duct and the presence of local inflammatory or infectious processes, such as a subphrenic or liver abscess [14, 15]. Clinically, the presence of bilioptysis is a pathognomonic sign of BBF [16, 17], and a definitive diagnosis can be made by ERC, bronchoscopy, or percutaneous transhepatic cholangiography [6, 14, 18]. Bilioptysis led us to suspect a BBF in our patient because it has been reported that bilioptysis is seen in all BBF patients. In our case, BBF was indeed diagnosed by fistulography from the tube that had been placed to drain the intraabdominal abscess. The pleural effusion drained after percutaneous abscess drainage did not contain bile or bacteria although it was possible that the spread of inflammation to the thoracic cavity via the intercostally inserted tube that had been used to drain the abscess might have accelerated the formation of the BBF. Therefore, in the present case, the inflammatory reaction in the subdiaphragmatic space due to bile leakage probably eroded the suture line of the diaphragm, which had been dissected during hepatectomy under the thoracoabdominal approach and the adherent lower lung with the diaphragm, leading to a communication between the biliary duct and bronchial tree.

**Table 1:** Review of reported cases with bronchobiliary fistula after treatments for hepatocellular carcinoma

Author	Age/sex	Location of HCC	Treatment for HCC	Bilioptysis	Biloma or abscess	Disorder of biliary Tree	Diagnostic modality of BBF	Treatment for BBF (prognosis)
Khandelwal	73/F	Dome	Chemotherapy	+	No	Stricture	ERCP	Biliary stent (5 months; died of cancer)
Akazawa	69/M	NA	TACE	+	Biloma	NA	None	Biliary stent (5 months; died of cancer)
Kaido	76/M	NA	Hr1(M)	+	Abscess	Stricture	99mTc-HIDA	PTAD and RML+RLL (12 days; died of hepatic failure)
Hibi	66/M	Right lobe	TACE	+	No	BTT	Bronchoscopy	Biliary stent and RHx+RLL+BR (6 months; alive)
Yoon	43/F	Lateral segment and dome	TACE and RFA	+	Abscess	NA	Fistulography	PTAD (2 months; alive)
Kim	52/F	Segment 7	RFA	+	Abscess	Stricture	CT	Hr1(P)+BR (2 months; alive)
Kuo	68/M	Segment 4	Central lobectomy	+	No	Stricture	Bronchoscopy	VATS (pneumolysis and resection of the BBF) (1 year; alive)
Dai	65/M	Posterior section	TACE+RFA	+	Abscess	NA	CT	Thoracic cavity drainage (1 day; died of respiratory failure)
Zhong	58/M	Segment 8	RFA	+	Biloma	Lithiasis	CT	RHx+lithotomy (18 months; alive)
Kim	53/M	Right lobe	TACE	+	Biloma	Stricture	Bronchoscopy and tubogram	Drainage and embolization of the BBF (1 month; died of hepatic failure)
Zeng	57/M	Segment 8	TACE+RFA	+	Biloma	Lithiasis	None	RHx+lithotomy (40 days; alive)
Present case	70/M	Segment 5, 7	Partial resection	+	Abscess	None	Fistulography	PTAD, ENBD⇒RHx (4 years; alive)

*NA* not available, *TACE* transcatheter arterial chemoembolization, *ERCP* endoscopic retrograde cholangiopancreatography, *Hr1*(*M*) medial segmentectomy, *PTAD* percutaneous transhepatic abscess drainage, *RML* right middle lobectomy of the lung, *RLL* right lower lobectomy of the lung, *BR* biliary reconstruction, *BTT* biliary tumor thrombus, *RHx* right hemihepatectomy, *RFA* radiofrequency ablation, *Hr1*(*P*) posterior sectionectomy, *VATS* video-assisted thoracoscopic surgery

A BBF is thought to be a serious complication associated with a high morbidity rate, including high rates of sepsis and pulmonary disorders, and often results in death [16]. Therefore, appropriate and prompt treatment is required for BBF, but there has been no consensus-based standard treatment. A few reports demonstrated that a BBF could be healed using only percutaneous drainage with the administration of antibiotics [7, 19]. However, most reported cases of BBF required additional treatment. Less-invasive procedures, such as endoscopic biliary drainage and placement of biliary stents have recently been employed in the treatment of BBF, especially in cases associated with biliary tract obstruction, because the endoscopic techniques have been improved. Surgical approaches with or without resection of the fistula tract and involved lung should be considered only after other intervention have failed [17, 20, 21]. BBF of our present case seemed to be not exactly healed only by continuous percutaneous drainage because bilioptysis was often seen, although bronchus was not demonstrated by fistulography. It was speculated that our patient could not recover from BBF as long as bile leakage was persistent.

Bile leakage is one of the most common complications after hepatic resection, and around 70% of the cases of bile leakages are thought to resolve spontaneously [22]. However, the bile leakage in the present case could not be healed by long-term percutaneous drainage. Several treatments, including ENBD and ethanol injection therapy, were attempted, but failed. Eventually, the nonsurgical procedures were concluded to be of limited use, and residual anterior sectionectomy of the liver, including the leaking ducts, was planned preoperatively based on volumetric analysis (ratio of estimated hepatic resection volume 22.1%) because BBF believed to be healed if bile leakage stopped. However, residual right bisectionectomy was actually performed because residual anterior sectionectomy might cause bile leakage although the patient had a risk of the postoperative hepatic failure due to major hepatectomy. Fortunately, the postoperative clinical course was good without bile leakage, bilioptysis, or pulmonary disorders.

## Conclusions

In conclusion, we experienced a rare case of BBF that occurred after hepatectomy for hepatocellular carcinoma. Careful attention should be paid to prevent bile leakage during hepatectomy since bile leakage has the potential to cause a BBF.

## References

[1] Jamal Y, Tombazzi C, Waters B, Ismail MK (2008). Bronchobiliary fistula in a cirrhotic patient: a case report and review of the literature. Am J Med Sci.

[2] Peacock TB (1850). Case in which hydatids were expectorated and one of suppuration of a hydatid cyst of the liver communicating with the lungs. Edin Med J.

[3] Khandelwal M, Inverso N, Conter R, Campbell D (1996). Endoscopic management of a bronchobiliary fistula. J Clin Gastroenterol.

[4] Akazawa S, Omagari K, Amenomori M, Nishiyama H, Mizuta Y, Kohno S (2004). Bronchobiliary fistula associated with intrahepatic biloma after transcatheter arterial chemoembolization for hepatocellular carcinoma. J Hepatol.

[5] Kaido T, Kano M, Suzaki S, Yanagibashi K, Shiota M (2006). Bronchobiliary fistula after hepatectomy for hepatocellular carcinoma. Dig Dis Sci.

[6] Hibi T, Sakamoto Y, Asamura H, Tochigi N, Ojima H, Shimada K (2007). Successful resection of hepatocellular carcinoma with bronchobiliary fistula caused by repeated transcatheter arterial embolizations: report of a case. Surg Today.

[7] Yoon DH, Shim JH, Lee WJ, Kim PN, Shin JH, Kim KM (2009). Percutaneous management of a bronchobiliary fistula after radiofrequency ablation in a patient with hepatocellular carcinoma. Korean J Radiol.

[8] Kim DH, Choi DW, Choi SH, Heo JS, Jeong J, Rhu J (2013). Surgical treatment of bronchobiliary fistula due to radiofrequency ablation for recurrent hepatocellular carcinoma. Korean J Hepatobiliary Pancreat Surg.

[9] Kuo YS, Lee SC, Chang H, Hsieh CB, Huang TW (2014). Thoracoscopic surgery for bronchobiliary fistula: a case report. J Cardiothorac Surg.

[10] Dai H, Cui D, Li D, Zhai BO, Zhang J (2015). Hepatic abscess with hepatobronchial fistula following percutaneous radiofrequency ablation for hepatocellular carcinoma: a case report. Oncol Lett.

[11] Zhong Y, Deng M, Li K, Xu R (2015). Delayed bronchobiliary fistula and cholangiolithiasis following percutaneous radio frequency ablation for hepatocellular carcinoma. Exp Biol Med (Maywood).

[12] Kim HY, Kwon SH, Oh JH, Shin JS, Dong SH, Park MJ (2016). Percutaneous transhepatic embolization of a bronchobiliary fistula developing secondary to a biloma after conventional transarterial chemoembolization in a patient with hepatocellular carcinoma. Cardiovasc Intervent Radiol.

[13] Zeng Z, Cai M, Huang W, Huang J, Chen X, Shan H (2016). Delayed bronchobiliary fistula following radiofrequency ablation in a patient with hepatocellular carcinoma: a case report and lesson regarding treatment. Oncol Lett.

[14] Gugenheim J, Ciardullo M, Traynor O, Bismuth H (1988). Bronchobiliary fistulas in adults. Ann Surg.

[15] Chua HK, Allen MS, Deschamps C, Miller DL, Pairolero PC (2000). Bronchobiliary fistula: principles of management. Ann Thorac Surg.

[16] Koch KA, Crump JM, Monteiro CB (1995). A case of biliptysis. J Clin Gastroenterol.

[17] Liao GQ, Wang H, Zhu GY, Zhu KB, Lv FX, Tai S (2011). Management of acquired bronchobiliary fistula: a systematic literature review of 68 cases published in 30 years. World J Gastroenterol.

[18] Oettl C, Schima W, Metz-Schimmerl S, Fugger R, Mayrhofer T, Herold CJ (1999). Bronchobiliary fistula after hemihepatectomy: cholangiopancreaticography, computed tomography and magnetic resonance cholangiography findings. Eur J Radiol.

[19] Cropper LD, Gold RE, Roberts LK (1982). Bronchobiliary fistula: management with percutaneous catheter drainage of a subphrenic abscess. J Trauma.

[20] Rose DM, Rose AT, Chapman WC, Wright JK, Lopez RR, Pinson CW (1998). Management of bronchobiliary fistula as a late complication of hepatic resection. Am Surg.

[21] Aydin U, Yazici P, Tekin F, Ozutemiz O, Coker A (2009). Minimally invasive treatment of patients with bronchobiliary fistula: a case series. J Med Case Rep.

[22] Vigano L, Ferrero A, Sgotto E, Tesoriere RL, Calgaro M, Capussotti L (2008). Bile leak after hepatectomy: predictive factors of spontaneous healing. Am J Surg.

